# Relationship Between Ischemic Heart Disease and Sexual Satisfaction

**DOI:** 10.5539/gjhs.v8n1p263

**Published:** 2015-06-10

**Authors:** Leila Ghanbari Afra, Mohsen Taghadosi, Hamid Reza Gilasi

**Affiliations:** 1 Faculty of Nursing and Midwifery, Kashan University of Medical Sciences, Kashan, Iran; 2 Department of Medical Surgical Nursing, Faculty of Nursing, Kashan University of Medical Sciences, Kashan, Iran; 3 Department of Epidemiology & Biostatistics, School of Health, Kashan University of Medical Sciences, Kashan, Iran

**Keywords:** sexual, satisfaction, ischemic heart disease, Iran

## Abstract

**Aim:**

Ischemic heart disease is a life-threatening condition. Considerable doubts exist over the effects of this disease on patients’ sexual activity and satisfaction. The aim of this study was to evaluate the relationship between ischemic heart disease and sexual satisfaction.

**Methods:**

In a retrospective cohort study, the convenience sample of 150 patients exposure with ischemic heart disease and 150 people without exposure it was drawn from Shahid Beheshti hospital, Kashan, Iran. Sampling was performed from March to September 2014. We employed the Larson’s Sexual Satisfaction Questionnaire for gathering the data. Data were analyzed using descriptive statistics and Chi-square, t-test and linear regression analysis.

**Results:**

The means of sexual satisfaction in patients exposure with ischemic heart disease and among the subjects without exposure it were 101.47±13.42 and 100.91±16.52, respectively. There was no significant difference between the two groups regarding sexual satisfaction. However, sexual satisfaction was significantly correlated with gender and the use of cardiac medications (P value < 0.05).

**Conclusion:**

The level of sexual satisfaction in patients with exposure ischemic heart disease is similar to the people without exposure it. Moreover, the men and the patients who do not receive cardiac medications have higher levels of sexual satisfaction. Nurses who are providing care to patients with ischemic heart disease need to pay closer attention to patient education about sexual issues.

## 1. Introduction

Sexual Satisfaction (SS) is defined as the pleasure at sexual relationship. SS improves the functions of families, facilitates parental role performance, enhances couples’ health, longevity, and life satisfaction, strengthens their emotional ties and marital relationships, and promotes their growth and development (Bakhshayesh & Mortazavi, 2010; Sarhadi et al., 2013). The importance of SS is so much critical that Maslow ranked it among the basic needs of human. Sometimes, SS is the most important factor behind the success of marital relationships (Vandermassen, 2004; Bakhshayesh & Mortazavi, 2010).

Ischemic heart disease (IHD) is a debilitating condition which causes different physical and psychosocial problems. Ineffective coping with IHD as well its associated problems may impair patients’ SS (Steinke et al., 2013; Levine et al., 2012). Factors such as vascular disorders, side effects of medications, and age-related changes in the functioning of body systems can compromise the functions of sex organs among patients with IHD (Camacho & Reyes-Ortiz, 2005; Jackson et al., 2006; Auslander et al., 2007; Bispo et al., 2013; Salonia et al., 2013). Moreover, psychological problems such as depression, anxiety, stress, and fear about developing new myocardial infarction (MI) or suffering death can also cause sexual dysfunction (Lukkarinen & Lukkarinen, 2007; Lunelli et al., 2008; Hazelton et al., 2009; Levine et al., 2012). Sexual impotence, lack of orgasm, as well as reduced frequency and quality of sexual relationships alter patients’ self-concept and worry them about the possibility of returning to normal levels of sexual activity (Lau et al., 2005; Dahabreh & Paulus, 2011; Eyada & Atwa, 2007).

Findings of studies on the relationship of heart problems and SS are conflicting. Although the likelihood of developing new MI or facing death during sexual intercourse is less than 1% (Dahabreh & Paulus, 2011; Levine et al., 2012; Steinke et al., 2013), but fear from negative effects of sexual activity on heart may cause avoidance of sexual relationship, anger towards one’s own health status, as well as sexual dissatisfaction (Lau et al., 2005; Pouraboli et al., 2009; Arenhall et al., 2011; Soderberg et al., 2013; Vazquez et al., 2010; Nascimento et al., 2013; McCall-Hosenfeld et al., 2008; Sarhadi et al., 2013). Lunelli et al. (2008) and Soderberg (2013) reported that the frequency of sexual relationships among patients with IHD is reduced by 40% to 70% (Nascimento et al., 2013; Lunelli et al., 2008). However, other studies reported that patients’ fear from facing death can promote their intimacy and improve their marital relationships, mutual understanding, and SS (Auslander et al., 2007; Bakhshayesh & Mortazavi, 2010; Bispo et al., 2013; Eyada & Atwa, 2007). Abramsohn et al. (2013) found that despite physical problems, patients with IHD resume their sexual activities four weeks after experiencing IHD (Abramsohn et al., 2013).

As mentioned, significant disagreement exists over the relationship between IHD and patients’ SS. Our field observations also confirmed differences in patients SS following IHD. Accordingly, this study was conducted to evaluate the relationship between IHD and SS.

## 2. Methods

### 2.1 Design and Patients

**Figure 1:**
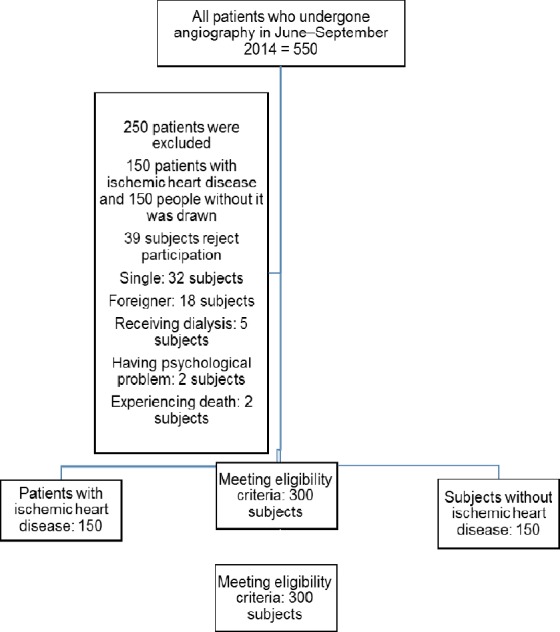
The flow diagram of the study

This study was conducted between March to September 2014 by using the retrospective cohort design. Study population was all the patients with IHD who had been hospitalized in the angiography unit of Shahid Beheshti hospital, Kashan, Iran. A convenience sample of 150 patients exposure with IHD and 150 people without exposure to it was drawn. The study sample size was calculated using the results of local study conducted by Taghadosi et al. Accordingly, with a type I and a type II error of respectively 0.05 and 0.80 and Based on the results of Taghadosi et al (2008), the sample size was determined to be 150 people for each group (Taghadosi & Gilasi, 2008). Participants who had a less-than-40% coronary occlusion were considered as healthy (without exposure to IHD) while participants whose coronary occlusion was greater than 40% were considered as having IHD (exposure with IHD) (Wiener et al., 2012) (Figure 1). The inclusion criteria were being married, giving informed consent for participation, having Iranian nationality, not having any known mental problems, being able to answer researchers’ questions and speak Persian, having no previous history of hospitalization due to cardiac problems, and being discharged maximally three days after hospitalization. Patients who wanted to withdraw from the study or were re-hospitalized were excluded.

### 2.2 Instruments

Study data were collected by using a demographic questionnaire (on participants’ age, gender, education, employment, use of cardiac medications, and history of other underlying diseases) and the Larson’s Sexual Satisfaction Questionnaire (LSSQ). The LSSQ is a 25-item standardized questionnaire for evaluating SS. Items are scored on a five-point Likert scale on which 1 is equal to ‘Never’ and 5 is equal to ‘Always’. Thirteen items are scored reversely. The total score of the LSSQ ranges from 25 to 125 (Mofaraheh Shams, Shah Siah, Mohebi, & Tabaraee, 2010, Larson, Anderson, Holman, & Niemann, 1998). The reliability and the validity of the Persian LSSQ were evaluated by Shams et al. (2010). They reported a Coronbach’s alpha of 0.98 for the questionnaire (Mofaraheh Shams et al., 2010).

### 2.3 Ethical Considerations

After receiving the necessary permissions and approvals, we referred to the study setting and identified eligible subjects. The aim and the methods of the study were explained to them and informed consent was obtained. Then, study subjects were invited to complete the demographic questionnaire. Two months afterward, subjects were invited to the study setting for completing the LSSQ. For subjects who were unable to read or write, questionnaires were filled by using the interview technique.

This study was conducted based on the Declaration of Helsinki and after being approved by the Institutional Review Board and the Ethics Committee of Kashan University of Medical Sciences, Kashan, Iran. We informed patients about the aim and the flow of the study and asked them to provide informed consent. Due to the great sensitivity of sexual issues and for preventing potential measurement biases, the questionnaires were administered and filled by same-gender questioners.

### 2.4 Data Analysis

We analyzed the data by using the SPSS v. 13.0. The similarity of the two groups regarding demographic variables—such as gender, education, history of underlying disease, and use of cardiac medications—was assessed by the Chi-square test. The independent-samples t-test was also employed for comparing the study groups in terms of age and SS. We also performed linear regression analysis for removing the confounding effects of intervening factors. P-Value less than 0.05 was considered significant in all analyses.

## 3. Results

In total, 550 subjects had been hospitalized in the study setting from whom 250 did not meet the inclusion criteria. Consequently, 300 subject—150 exposure with IHD and 150 without exposure to it—entered and completed the study. The Chi-square test revealed that the two groups did not differ significantly regarding education (P value = 0.06; Table 1). However, the results of the Chi-square and the independent-samples t-tests showed that there were significant differences between the two groups in terms of gender, age, employment, history of chronic diseases, and use of cardiac medications (P value < 0.05).

**Table 1:** Study participants’ demographic and clinical characteristics

Groups Variables		Exposure with IHD		Exposure without IHD		P

		N	%	N	%	
Gender	Male	106	70.7	**67**	44.7	0.000
	Female	44	29.3	83	55.3	
Education	Illiterate	112	74.7	97	64.7	0.06
	Literate	38	25.3	53	35.3	
Employment	unemployed	90	60	108	72	0.03
	Employed	60	40	42	28	
history of underlying disease	Yes	125	83.3	97	64.7	0.000
	No	25	16.7	53	35.3	
Use of cardiac medications	Yes	108	72	73	48.7	0.000
	No	42	28	77	51.3	

The means of SS in subjects with and without exposure to IHD were 101.47±13.42 and 100.91±16.52, respectively. The independent-samples t-test indicated that study groups did not differ significantly concerning the levels of SS (P value = 0.869). About 70.7% of patients with IHD and 64% of healthy subjects were highly satisfied with their sexual relationships (P value = 0.4). We conducted linear regression analysis to remove the confounding effects of the variables of gender, age, education, employment, history of chronic disease, and use of cardiac medications. The results of linear regression analysis revealed that there was no significant relationship between IHD and SS after controlling the confounding variables (P value = 0.74).

## 4. Discussion

The aim of this study was to compare the level of SS in people with and without exposure to IHD. Study findings showed that two months after experiencing IHD, SS rate was similar in both groups. Abramsohn et al. (2013) also reported that despite a significant decrease in the frequency of sexual relationship among American women with MI, couples had intimate marital relationships and high SS. They found that the participating women had resumed their sexual activities four weeks after experiencing MI (Abramsohn et al., 2013). The findings of another study conducted by Lukkarinen and Lukarinen (2007) in Finland also revealed that after administrating medical and surgical treatments (medications, coronary angioplasty, and coronary bypass grafting), the level of SS did not differ significantly (Lukkarinen & Lukkarinen, 2007). However, Soderberg (2013) and Eyada and Atwa (2007) found that unstable angina as well as ST-elevation and non-ST-elevation MIs significantly affected SS (Eyada & Atwa, 2007; Soderberg et al., 2013). Padash and Abedi (2012) also found that there was a significant difference between patients with coronary disease and healthy people regarding SS (Nascimento et al., 2013). It seems that increased coronary blood flow and improved functional capacity following medical and surgical treatments and nursing care improve patients’ tolerance and SS (Levine et al., 2012; Lukkarinen & Lukkarinen, 2007). Moreover, an acute illness such as IHD both reduces patients’ sexual needs and requires family members to actively support them. Accordingly, the SS for patients with IHD can be similar to that of the healthy individuals (Arenhall et al., 2011). The discrepancies between our findings and the findings of Soderberg (2013), Eyada and Atwa (2007), Padash and Abedi (2012), and Nacimento et al. (2013) can be attributed to the differences in the samples and the designs of the studies. Eyada and Atwa (2007) conducted a quantitative study on 35 women while Soderberg (2013) used a qualitative approach for exploring eleven women’s experiences of post-MI sexual health. In other words, the samples of these two studies consisted of only women while we studied both men and women. Other possible reasons for such discrepancies can be the differences in the instruments and the settings of the studies. Sarhadi et al. (2013) noted that cultural beliefs in different communities can affect the findings of studies on sexual issues.

We also found that compared with women, the participating men had a significantly higher SS-98.94±18.92 vs. 102.84±11.12, respectively. Eyada and Atwa (2007), Kazemi et al. (2008), Lukkarinen and Lukarinen (2007), and Soderberg (2013) also reported the same finding (Eyada & Atwa, 2007; Kazemi-Saleh et al., 2008; Lukkarinen & Lukkarinen, 2007; Soderberg et al., 2013). This finding can be attributed to the more severe age-related physiologic and hormonal changes among women. Estrogen affects vaginal blood flow and decreases vaginal secretions which in turn causes dyspareunia and postpones women’s sexual arousal. Moreover, women’s SS is highly dependent on psychological factors such as feelings of intimacy, love, and interest. Women usually find great SS in courtship and necking. However, as men usually reach orgasm in relatively shorter times, women’s need to courtship and necking often remains unfulfilled and hence, their SS rate is lower than men (Soderberg et al., 2013; Steinke et al., 2013; McCall-Hosenfeld et al., 2008; Lukkarinen & Lukkarinen, 2007; Levine et al., 2012; Abramsohn et al., 2013).

Study findings also revealed that the subjects who did not receive cardiac medications felt higher SS than the patients who took such medications. The mean SS scores of these two groups were 104.29±11.05 and 99.15±16.86, respectively. Lukkarinen (2007) also found that patients who received cardiac medications had lower levels of SS. Nitrates, beta-blockers, and diuretics decrease libido, postpone or prevent orgasm, and negatively affect SS (Levine et al., 2012; Steinke et al., 2013).

## 5. Conclusions

Study findings indicated that two months after experiencing IHD, patients’ level of SS was similar to subjects without IHD. Moreover, SS was lower among females as well as the patients who received cardiac medications. Nurses who are providing care to patients with IHD need to pay closer attention to patient education about sexual issues.

**Study Limitations**

This study had two limitations. First, patients hold different religious and cultural beliefs and hence, may have different levels of SS. In addition, they have different physiologic conditions. These two limitations might have affected the study findings.

## References

[ref1] Abramsohn E. M, Decker C, Garavalia B, Garavalia L, Gosch K, Krumholz H. M, Spertus J. A, Lindau S. T (2013). “I'm Not Just a Heart, I'm a Whole Person Here”: A Qualitative Study to Improve Sexual Outcomes in Women With Myocardial Infarction. Journal of the American Heart Association.

[ref2] Arenhall E, Kristofferzon M.-L, Fridlund B, Malm D, Nilsson U (2011). The male partners' experiences of the intimate relationships after a first myocardial infarction. European Journal of Cardiovascular Nursing.

[ref3] Auslander B. A, Rosenthal S. L, Fortenberry J. D, Biro F. M, Bernstein D. I, Zimet G. D (2007). Predictors of sexual satisfaction in an adolescent and college population. Journal of Pediatric and Adolescent Gynecology.

[ref4] Bakhshayesh A, Mortazavi M (2010). The relationship between sexual satisfaction, general health and marital satisfaction in couples. Journal of Applied Psychology.

[ref5] Bispo G. S, De Lima Lopes J, De Barros A. L (2013). Cardiovascular changes resulting from sexual activity and sexual dysfunction after myocardial infarction: integrative review. Journal of Clinical Nursing.

[ref6] Camacho M, Reyes-Ortiz C (2005). Sexual dysfunction in the elderly: age or disease?. International journal of impotence research.

[ref7] Dahabreh I. J, Paulus J. K (2011). Association of Episodic Physical and Sexual Activity With Triggering of Acute Cardiac Events Systematic Review and Meta-analysis. JAMA.

[ref8] Eyada M, Atwa M (2007). Sexual function in female patients with unstable angina or non-ST-elevation myocardial infarction. The journal of sexual medicine.

[ref9] Hazelton A. G, Sears S. F, Kirian K, Matchett M, Shea J (2009). Coping with my partner, ICD and cardiac disease. Circulation.

[ref10] Jackson G, Rosen R. C, Kloner R. A, Kostis J. B (2006). Report: The Second Princeton Consensus on Sexual Dysfunction and Cardiac Risk: New Guidelines for Sexual Medicine. The journal of sexual medicine.

[ref11] Kazemi-Saleh D, Pishgou B, Farrokhi F, Assari S, Fotros A, Naseri H (2008). Gender impact on the correlation between sexuality and marital relation quality in patients with coronary artery disease. The journal of sexual medicine.

[ref12] Larson J. H, Anderson S. M, Holman T. B, Niemann B. K (1998). A longitudinal study of the effects of premarital communication, relationship stability and self-esteem on sexual satisfaction in the first year of marriage. J Sex Marital Ther.

[ref13] Lau J. T, Kim J. H, Tsui H. Y (2005). Mental health and lifestyle correlates of sexual problems and sexual satisfaction in heterosexual Hong Kong Chinese population. Urology.

[ref14] Levine G. N, Steinke E. E, Bakaeen F. G, Bozkurt B, Cheitlin M. D, Conti J. B, Lange R. A (2012). Sexual Activity and Cardiovascular Disease A Scientific Statement From the American Heart Association. Circulation.

[ref15] Lukkarinen H, Lukkarinen O (2007). Sexual satisfaction among patients after coronary bypass surgery or percutaneous transluminal angioplasty: Eight-year follow-up. Heart & Lung: The Journal of Acute and Critical Care.

[ref16] Lunelli R. P, Rabello E. R, Stein R, Goldmeier S, Moraes M. A (2008). Sexual activity after myocardial infarction: taboo or lack of knowledge?. Arq Bras Cardiol.

[ref17] Mccall-Hosenfeld J. S, Freund K. M, Legault C, Jaramillo S. A, Cochrane B. B, Manson J. E, Rodriguez B. L (2008). Sexual satisfaction and cardiovascular disease: The women's Health Initiative. The American journal of medicine.

[ref18] Mofaraheh Shams Z, Shah Siah M, Mohebi S, Tabaraee Y (2010). The effect of marital counseling on sexual satisfaction of couples in Shiraz city. Journal of Health System.

[ref19] Nascimento E. R, Maia A. C, Pereira V, Soares-Filho G, Nardi A. E, Silva A. C (2013). Sexual dysfunction and cardiovascular diseases: a systematic review of prevalence. Clinics.

[ref20] Pouraboli B, Azizzadeh Foruzi M, Mohammad Alizade A (2009). Knowledge and attitudes of nurses in sexual activity and educate it to patients with myocardial infarction and their spouses. Iran J Crit Care Nurs.

[ref21] Salonia A, Capogrosso P, Clementi M. C, Castagna G, Damiano R, Montorsi F (2013). Is erectile dysfunction a reliable indicator of general health status in men?. Arab Journal of Urology.

[ref22] Sarhadi M, Navidian A, Fasihi Harandy T, Ansari Moghadam A. R (2013). Comparing quality of marital relationship of spouses of patients with and without a history of myocardial infarction. Journal of Health Promotion Management.

[ref23] Soderberg L. H, Johansen P. P, Herning M, Berg S. K (2013). Women's experiences of sexual health after first-time myocardial infarction. J Clin Nurs.

[ref24] Steinke E. E, Jaarsma T, Barnason S. A, Byrne M, Doherty S, Dougherty C. M, Mosack V (2013). Sexual counselling for individuals with cardiovascular disease and their partners A Consensus Document From the American Heart Association and the ESC Council on Cardiovascular Nursing and Allied Professions (CCNAP). European heart journal.

[ref25] Taghadosi M, Gilasi H. R (2008). The general and specific quality of life in patients with Ischemia in Kashan. Iranian Journal Of Nursing Research.

[ref26] Vandermassen G (2004). Sexual Selection: A tale of male bias and feminist denial. European Journal of Women's Studies.

[ref27] Vazquez L. D, Sears S. F, Shea J. B, Vazquez P. M (2010). Sexual health for patients with an implantable cardioverter defibrillator. Circulation.

[ref28] Wiener C, Fauci A, Braunwald E, Kasper D, Hauser S, Longo D, Brown C (2012). Harrisons Principles of Internal Medicine Self-Assessment and Board Review.

